# RNA-Seq analysis on chicken taste sensory organs: An ideal system to study organogenesis

**DOI:** 10.1038/s41598-017-09299-7

**Published:** 2017-08-22

**Authors:** Xiaogang Cui, Brett Marshall, Ning Shi, Shi-You Chen, Romdhane Rekaya, Hong-Xiang Liu

**Affiliations:** 1 Regenerative Bioscience Center, University of Georgia, Athens, GA USA; 2 Department of Animal and Dairy Science, College of Agricultural and Environmental Sciences, University of Georgia, Athens, GA USA; 3 Department of Physiology and Pharmacology, College of Veterinary Medicine, University of Georgia, Athens, GA USA; 4 Institute of Bioinformatics, University of Georgia, Athens, GA USA

## Abstract

RNA-Seq is a powerful tool in transcriptomic profiling of cells and tissues. We recently identified many more taste buds than previously appreciated in chickens using molecular markers to stain oral epithelial sheets of the palate, base of oral cavity, and posterior tongue. In this study, RNA-Seq was performed to understand the transcriptomic architecture of chicken gustatory tissues. Interestingly, taste sensation related genes and many more differentially expressed genes (DEGs) were found between the epithelium and mesenchyme in the base of oral cavity as compared to the palate and posterior tongue. Further RNA-Seq using specifically defined tissues of the base of oral cavity demonstrated that DEGs between gustatory (GE) and non-gustatory epithelium (NGE), and between GE and the underlying mesenchyme (GM) were enriched in multiple GO terms and KEGG pathways, including many biological processes. Well-known genes for taste sensation were highly expressed in the GE. Moreover, genes of signaling components important in organogenesis (Wnt, TGFβ/ BMP, FGF, Notch, SHH, Erbb) were differentially expressed between GE and GM. Combined with other features of chicken taste buds, e.g., uniquely patterned array and short turnover cycle, our data suggest that chicken gustatory tissue provides an ideal system for multidisciplinary studies, including organogenesis and regenerative medicine.

## Introduction

RNA sequencing (RNA-Seq) technology has emerged as a powerful and revolutionary approach to quantify gene expression levels and survey detailed transcriptomic profiling at unprecedented resolution and sensitivity^1, 2^. Additionally, it is an invaluable tool for gene discovery^3, 4^. In comparison to microarray platforms, RNA-Seq has several advantages, including a wider dynamic range of expression levels, higher accuracy and reproducibility, and lower noise-to-signal ratio, resulting in an enhanced ability to detect novel transcripts^2, 5^. Consequently, RNA-Seq has attracted broad interest and led to significant breakthroughs in our understanding of the genetic and molecular basis of living organisms, including traits of economic interest in livestock species^6–16^.

Chickens (Gallus sp.) are widely used as a research model in multidisciplinary studies including developmental biology^17, 18^, molecular biology^19–22^, and food science^23^. Their popularity stems from their comparative advantages over other animal models, including the convenience of *in ovo* embryo manipulation, rapid development, high availability and low costs. Similarly to mammals, chickens have many taste buds in the oral cavity and respond to taste stimuli^24–27^. Our recent studies using molecular markers to label chicken taste buds in oral epithelial sheets, i.e., palate, base of the oral cavity and posterior region of the tongue, demonstrated that chicken taste buds, like those of mammals, are distributed in a unique pattern^28^. In peeled chicken oral epithelial sheets, taste buds labeled with *Vimentin* and *α-Gustducin* were easily identified. Many more taste buds, patterned in rosette-like clusters, were found than previously reported^28^ suggesting that chickens possess a more advanced taste system than previously believed. Moreover, the clustered taste bud patterning in the oral cavity of chickens is reminiscent of the mammalian soft palate^29^. Taken together, these data suggest that taste sensory organs in chickens can potentially provide a system for organogenesis studies, including pattern formation.

To better understand the transcriptomic architecture of gustatory tissue in the oral cavity of chickens, RNA-Seq analysis was carried out with the following specific objectives: i) to demonstrate the validity of chicken taste organs as an ideal system for organogenesis studies, ii) to provide new insights into the underlying mechanisms implicated in the development of taste buds. Such information will facilitate studies on mechanisms underlying chicken taste bud formation which will be beneficial for understanding taste organ development in birds and potentially mammals, including humans.

## Materials and Methods

### Animal and tissue collection

The use of animals throughout the study was approved by The University of Georgia Institutional Animal Care and Use Committee and was in compliance with the National Institutes of Health Guidelines for the care and use of animals in research.

Newly hatched Cobb 500 (P0) broiler-type male chickens were provided by Cobb-Vantress Inc. from its hatchery in Cleveland, Georgia. The chicks were housed in separate cages in the animal facility at the Department of Animal and Dairy Science, University of Georgia until 3 days of age (P3). The brooder temperature was ~35°C and room temperature was maintained at 30°C with food (starter feed) and water available *ad libitum* under a 12/12 hr light/dark cycle.

P3 chicks (n = 3) were euthanized by decapitation. The oral tissue in the palate, base of the oral cavity, and posterior region of the tongue were dissected and processed for RNA extraction. To separate the epithelium from the underlying connective tissue, the upper and lower beak were dissected and briefly rinsed in 0.1 M phosphate buffered saline (PBS). An enzyme mixture of Collagenase A (1 mg/ml, Cat# 10103578001, Roche Diagnostics) and Dispase II (2.5 mg/ml, Cat# 04942078001, Roche Diagnostics) was injected (~6 ml in total) into the sub-epithelial space of the palate, the base of the oral cavity, and the posterior region of the tongue, followed by incubation at 37°C for 2 hr. Following enzymatic tissue digestion, the palate, the base of the oral cavity and the posterior region of the tongue were dissected in sterile PBS, and epithelial sheets were separated from underlying connective tissue.

To collect gustatory and non-gustatory tissues at the base of the oral cavity, the soft tissue of the base of oral cavity was cut off from the lower beak. Tissues with taste buds were easy to recognize under a stereomicroscope and they were separated from the surrounding region lacking taste buds. The epithelium and underlying connective tissue were further separated for these two regions (with and without taste buds) and the following tissue compartments were collected: gustatory epithelium (GE), gustatory mesenchyme/connective tissue (GM), non-gustatory epithelium (NGE), non-gustatory mesenchyme/connective tissue (NGM).

### RNA isolation and quality assessment

Freshly isolated tissues were immersed in Trizol and immediately stored at −80°C until time of RNA extraction. Upon harvest of all tissues, samples were thawed on ice and homogenized with a PowerGen 700D tissue homogenizer (Fisher Scientific, Waltham, MA). The resulting homogenate was then processed via RNeasy Plus kit (Qiagen, Hilden, Germany), omitting the gDNA eliminator step, and was reconstituted in RNAse-free water. Initial RNA quantity and quality were determined on a Nanodrop 8000 spectrophotometer (Nanodrop, Thermo Scientific, Waltham, MA), and high quality samples were further assessed on an Agilent 2100 Bioanalyzer (Agilent Technologies, Santa Clara, CA) at the University of Georgia Genomics Facility (Athens, GA).

### cDNA library preparation, pooling, and sequencing

cDNA libraries for each sample were prepared with Kapa Stranded mRNA-seq kit (KAPA Biosystems, Wilmington, MA). Library quality and quantity were assessed via Fragment Analyzer Automated CE (Advanced Analytical, Evry Cedex, France) and Qubit (Thermo Fisher) systems, respectively. Libraries were subsequently subjected to 2 × 75 bp paired-end (PE75) sequencing on a NextSeq. 500 system (Illumina). All samples were pooled and sequenced on one lane. All library preparation and sequencing were performed by the Georgia Genomics Facility (Athens, GA).

### Reads alignment to the chick reference genome and annotated transcripts

To ensure high-quality data, reads with low-quality, containing adapter contamination, or at least 10 Ns from raw data (FASTQ format) were removed using in-house developed Perl scripts. Prior to downstream analysis, the overall quality of pre-processed data was further examined using FastQC v0.11.4 (http://www.bioinformatics.babraham.ac.uk/projects/fastqc/). The Galgal4 reference assembly (FASTA format) and annotated gene model (GTF format) were downloaded from Ensembl database (http://ftp//ftp.ensembl.org/pub/release-76/). For each library, we estimated the actual insert size distribution after indexing the reference genome using Bowtie2 v2.2.4 with default parameters^30^. Subsequently, paired-end clean reads were aligned to the reference genome using Tophat v2.0.14 (http://tophat.cbcb.umd.edu/)^31^. The detailed alignment information is presented in Supplemental Table S1, including total numbers of reads, mapped reads, and unique mapped reads.

### Identification of differentially expressed genes (DEGs)

The number of RNA-Seq reads produced by a transcript is directly proportional to its abundance. Thus, the gene expression level could be easily quantified by the read count. Cuffdiff v2.2.1^32^ and DESeq2 R packages^4, 33^, the main statistical methods to identify DEGs across different experimental conditions, were used.

For Cuffdiff, commonly used fragments were normalized by the relevance to the transcript’s length and the total yield of the fragments to ensure accurate quantification of each gene’s expression. The obtained values are represented by the number of fragments per kilobase of transcript per a million of mapped fragments (FPKM)^34^ in paired-end sequencing experiments^32^. TopHat’s read alignments were assembled by Cufflinks^32^, and then the DEGs and transcripts between two groups were detected and quantified by Cuffdiff using a rigorous sophisticated statistical analysis^32^. The corresponding attributes including fold changes, *p*-values, and *q*-values (false discovery rate corrected *p* values) of DEGs were reported in the output files from Cuffdiff. The difference in gene expression was determined as significant if the *p*-value was less than the false discovery rate after Benjamini-Hochberg correction for multiple testing (http://cufflinks.cbcb.umd.edu/manual.html).

For DESeq2 method, DEGs were detected using the DESeq2 R/Bioconductor package that performs independent filtering. To enhance the statistical power for identifying DEGs, we removed genes with weak expression levels using the HTS Filter package^35^. RNA-Seq read counts were analyzed using a generalized linear model for comparisons focusing on the GE vs NGE and GE vs GM. The resulting *p*-values were adjusted using Benjamini and Hochberg’s approach for controlling for false discovery rate. The fold changes, *p*-values and *q*-values (false discovery rate corrected *p*-values) of the DEGs were reported in the output files from DESeq2. Genes with a *q*-value <0.05 were assumed as differentially expressed.

### GO and gene functional analysis of differentially expressed genes

To gain insight into the biological functions of DEGs, the enriched Gene Ontology (GO) terms and Kyoto Encyclopedia of Genes and Genomes (KEGG) pathways were analyzed using Database for Annotation, Visualization and Integrated Discovery (DAVID) Bioinformatics Resources 6.8 (https://david.ncifcrf.gov/). The functional groups with at least two DEGs in the background terms were selected and those with a *p*-value < 0.05 were considered as significantly overrepresented.

### Real-time quantitative RT-PCR (qRT-PCR) assay

To confirm sequencing results, qRT-PCR on 14 randomly selected DEGs from GE vs NGE and 14 DEGs from GE vs GM was performed with the same RNA samples used for RNA-Seq. Primers were designed via Primer Express 3.0.1 software (Applied Biosystems) and are shown in Supplemental Table S2 (GE vs NGE) and Supplemental Table S3 (GE vs GM). Briefly, 1 μg of total RNA was reverse-transcribed to cDNA using the SuperScript First Strand cDNA conversion kit (Invitrogen, Carlsbad, CA). qRT-PCR was subsequently carried out in duplicate 10 μl reactions using SYBR green master mix (Genecopoeia, Rockville, MD). Reactions were run in a 96-well plate using an MX3000 P system (Agilent Technologies). The cycle conditions were as follows: 1 cycle of pre-incubation at 95°C for 10 min, 40 cycles of amplification (95°C for 30 s, 63°C for 15 s, and 72°C for 20 s). Relative gene expressions of DEGs were calculated using 2^−ΔΔCt^ method, with the housekeeping gene *GAPDH* serving as internal control.

## Results

### More differentially expressed genes (DEGs) between epithelium and connective tissue of the base of oral cavity than palate and tongue

**Figure 1:**
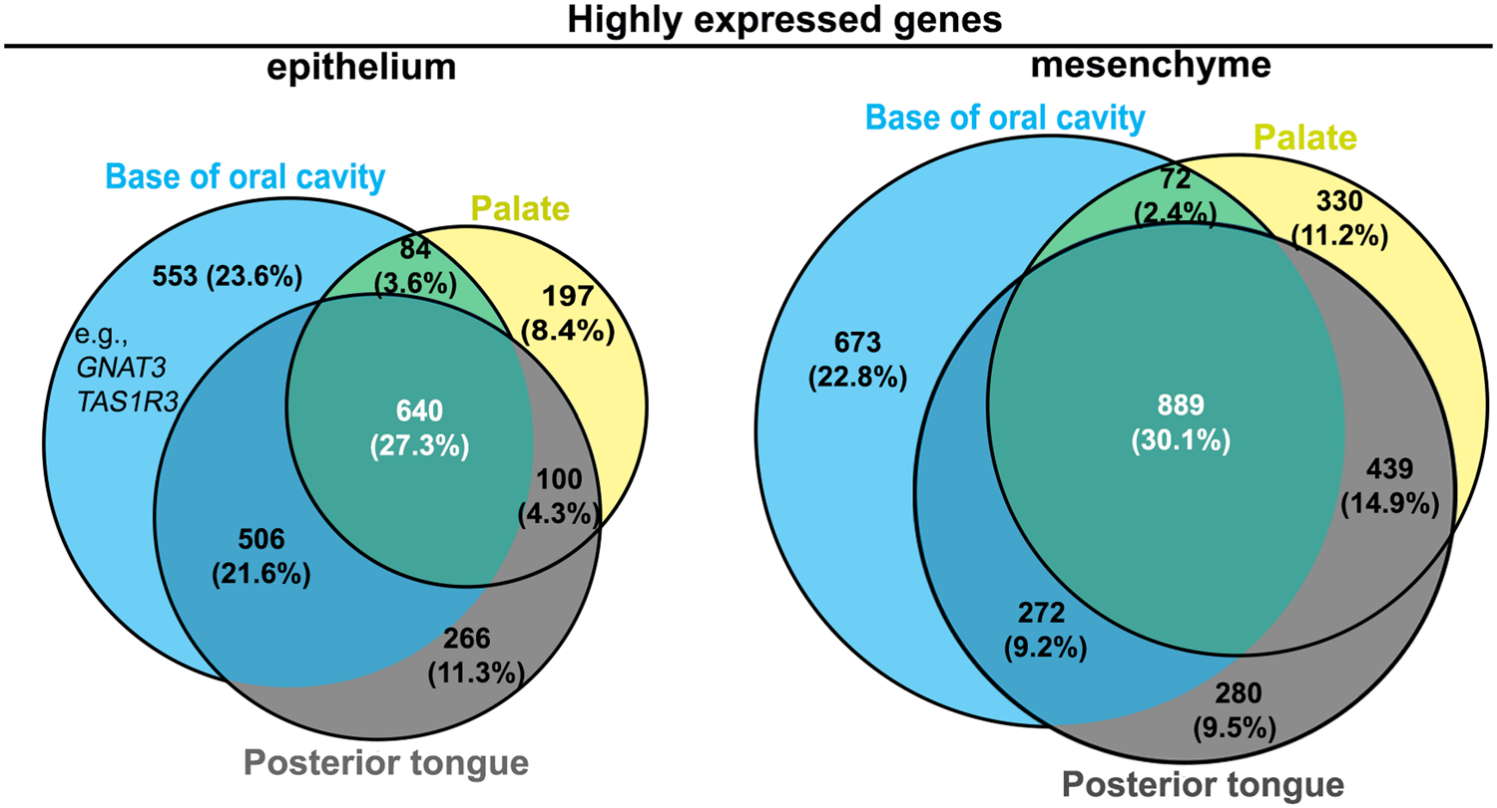
Number and overlapping of DEGs between epithelium and underlying mesenchyme of the palate, base of oral cavity, and posterior tongue in P3 male chickens. The highly expressed genes in the oral epithelium (left) or the underlying mesenchyme (right) in different tissues were analyzed separately followed by further comparisons of highly DEGs in the epithelium (left) or mesenchyme (right) of different tissues. The DEGs were determined by statistical algorithms Cuffdiff and DESeq2. Notably, the base of oral cavity had a greater number of highly expressed DEGs (*p* < 0.05) in both the epithelium and mesenchyme compared to the palate and posterior tongue. Genes that are well known to be involved in taste perception, e.g., *GNAT3*, *TAS1R3*, were revealed as highly DEGs in the epithelium of the base of oral cavity.

In the first RNA-Seq experiment, the entire epithelial sheet and underlying connective tissues of the palate, base of oral cavity, and posterior tongue were used. We demonstrated that (1) more DEGs were found between the epithelium (1783 highly expressed) and connective tissue (1906 highly expressed) in the base of the oral cavity (Supplemental Table S4) than palate or posterior tongue (1021 highly expressed in the epithelium and 1730 in the connective tissue of palate; 1512, 1880 in the posterior tongue, listed in Supplemental Tables S5 and S6, respectively); (2) a large overlap of DEGs (640 and 889) between epithelium and connective tissues of the palate, base of oral cavity, and posterior tongue were observed using DESeq2 and Cuffdiff (Fig. 1).

More importantly, DEGs that are associated with taste perception were found in the base of oral cavity, e.g., *GNAT3*, *TAS1R3* (Fig. 1, Supplemental Table S4), but were not detected in the palate and posterior tongue (Supplemental Tables S5, S6). In addition, our recent studies have demonstrated that taste bud clusters in the base of oral cavity are more uniformly distributed than those in the palate and tongue^28^. Further, the epithelium of the base of oral cavity was easy to separate from underlying tissues^28^, and the topography and tissue structure are less complex, e.g., no papillary spines. Based on these results, we focused our interest on the base of the oral cavity. Tissue samples from the gustatory epithelium (GE, epithelial region that contains taste buds), the non-gustatory epithelium (NGE, epithelium that surrounds the taste bud region), and the corresponding underlying connective tissue/mesenchyme (GM and NGM) of the base of the oral cavity were collected and used for additional RNA-Seq analyses.

### DEGs between gustatory epithelium and surrounding tissue compartments (NGE and GM)

Comparisons were focused on GE vs NGE and vs GM, all in triplicate, resulting in nine total tissues. We acquired a total of 335.66 million raw 100-bp paired-end reads. After quality filtering, the RNA-Seq of nine samples yielded around 325.59 million of clean reads with an average of 36.18 million reads per sample. Alignment of the sequence of clean reads against Galgal4 yielded 80.26–85.65% uniquely aligned reads across the nine samples using Tophat2 aligner. Furthermore, 96.55–98.70% of the reads were aligned in a unique manner, while the remaining were multiple-mapped reads. Detailed information of data quality and mapping statistics are listed in Supplemental Table S1.

**Figure 2:**
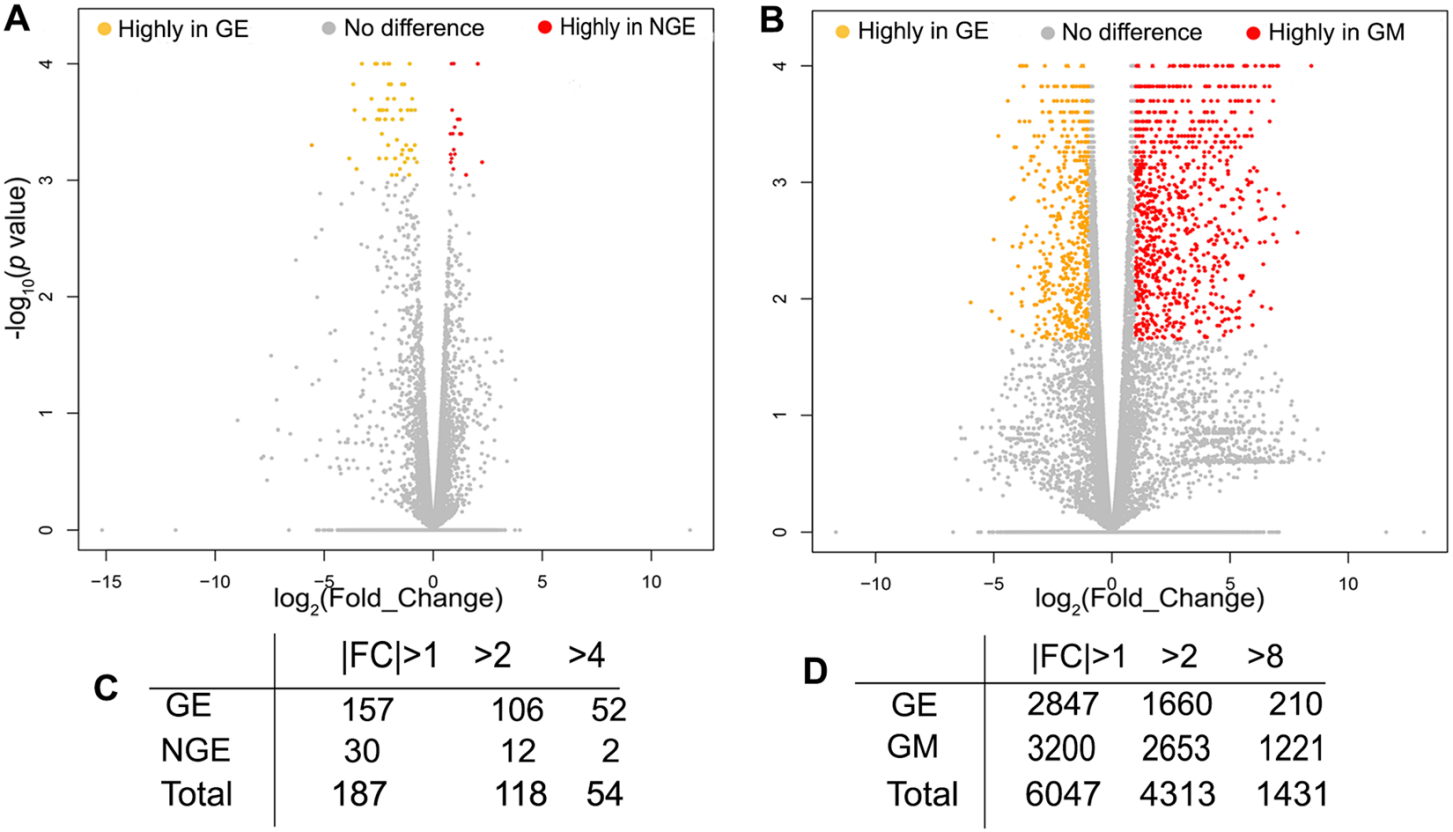
Volcano plots (**A**,**B**) and number of highly expressed DEGs at different fold changes (FC) (**C**,**D**) to display the DEGs in the GE vs NGE (**A**,**C**) and GE vs GM (**B**,**D**) in the base of oral cavity of P3 male chickens. In A and B, the Y-axis corresponds to the mean expression value of log10 (*p*-Value), and the X-axis displays the log2 (Fold_Change). The orange and red dots represent highly expressed genes in the GE (**A**,**B**) or NGE (**A**) or GM (**B**) (*p* < 0.05, FDR *q* < 0.05); the gray dots represent the transcripts whose difference of expression levels did not reach statistical significance (*p* > 0.05, FDR *q* > 0.05). GE: gustatory epithelium; NGE: non-gustatory epithelium; GM: gustatory mesenchyme.

In order to identify candidate genes involved in the development of taste buds in the gustatory epithelium (GE), Cufflinks and DESeq2 were used to identify the DEGs between GE vs NGE, and GE vs GM. A total of 187 and 6047 genes were differentially expressed between the GE and NGE and between GE and GM, respectively, based on |FC| > 1, *p* < 0.05, and FDR *q* < 0.05 detected by Cuffdiff or DESeq2 or both. Volcano plots of DEGs between the different tissue compartments are presented in Fig. 2.

Between GE and NGE, 118 genes were highly differentially expressed with a |FC| > 2, *p* < 0.05, and FDR *q* < 0.05, of which 106 and 12 genes were highly expressed in GE and NGE, respectively (Fig. 2C). Additionally, 65 of these 118 highly DEGs were identified by both Cuffdiff and DESeq2 methods (only 24 and 29 DEGs were identified by DESeq2 or Cuffdiff alone respectively). A much larger number of DEGs was identified between GE and GM using the same significance criteria. In fact, 4313 genes were differentially expressed with 1660 and 2653 highly expressed in the GE and GM, respectively (Fig. 2D). Almost 60% (2584) of these 4313 genes were identified by both pieces of software used in the analyses, and only 981(23%) and 748 (17%) were detected by either DESeq2 or Cuffdiff Approach alone.

The top 10 highly expressed genes in the GE or NGE based on the GE vs NGE comparison are listed in Table 1. The top 10 highly expressed genes in the GE or GM according to the GE vs GM comparison are presented in Table 2. Detailed information (Gene ID, Gene name, Fold_Change, *p*_value, *q*_value) of all DEGs are presented in Supplemental Table S7 (GE vs NGE) and Supplemental Table S8 (GE vs GM).

**Table 1:** Top 10 DEGs in GE and in NGE in comparison between GE and NGE.

Highly expressed genes in the GE					Highly expressed genes in the NGE				
Gene ID	Name	Fold Change	*p*_value	*q_*value	Gene ID	Name	Fold Change	*p*_value	*q*_value
ENSGALG00000004083	*GRIA1*	47.60	6.82E-07	1.50E-04	ENSGALG00000007815	*ANKK1*	4.87	3.84E-09	1.49E-06
ENSGALG00000010163	*LGR5*	28.59	2.34E-22	7.58E-19	ENSGALG00000019551	*OVALY*	4.12	1.00E-04	8.16E-03
ENSGALG00000011633	*SLC5A8*	25.42	1.54E-10	7.87E-08	ENSGALG00000008909	*USP13*	3.51	1.20E-07	3.07E-05
ENSGALG00000020084	*CAPN13*	21.17	5.00E-05	4.43E-03	ENSGALG00000000619	*ANGPTL4*	3.15	5.00E-05	4.43E-03
ENSGALG00000014884	*ISL1*	20.83	6.68E-48	6.49E-44	ENSGALG00000039830	*DHX30*	2.86	5.00E-05	4.43E-03
ENSGALG00000016325	*GSTA3*	19.46	6.03E-20	1.46E-16	ENSGALG00000000544	*RAB44*	2.84	9.00E-04	4.90E-02
ENSGALG00000016943	*OLFM4*	13.13	5.00E-05	4.43E-03	ENSGALG00000008759	*LAMP3*	2.78	7.43E-07	1.64E-04
ENSGALG00000011962	*NDNF*	12.67	3.53E-10	1.71E-07	ENSGALG00000041114	*GOLGA4*	2.35	5.00E-05	4.43E-03
ENSGALG00000016428	*ENPP2*	12.30	3.16E-09	1.39E-06	ENSGALG00000013994	*TAAR1*	2.35	4.00E-04	2.56E-02
ENSGALG00000026246	*GIF*	12.17	2.61E-07	6.33E-05	ENSGALG00000011687	*AHNAK2*	2.17	3.00E-04	2.00E-02

**Table 2:** Top 10 DEGs in GE and in GM in comparison between GE and GM.

Highly expressed genes in the GE					Highly expressed genes in the GM				
Gene ID	Name	Fold Change	*p*_value	*q*_value	Gene ID	Name	Fold Change	*p*_value	*q*_value
ENSGALG00000000341	*KRTAP10-4*	41.53	1.05E-04	2.94E-04	ENSGALG00000033157	*ADAMTS10*	316.56	5.00E-05	2.14E-04
ENSGALG00000007815	*ANKK1*	35.58	1.68E-08	8.02E-08	ENSGALG00000005643	*LYVE1*	316.14	1.75E-39	1.17E-37
ENSGALG00000035166	*LYPD2*	24.80	5.00E-05	2.14E-04	ENSGALG00000010391	*MMRN1*	302.00	1.28E-70	6.17E-68
ENSGALG00000039572	*PADI1*	23.01	5.00E-05	2.14E-04	ENSGALG00000022815	*AvBD1*	252.53	3.39E-18	4.53E-17
ENSGALG00000010864	*EREG*	22.94	5.31E-24	1.14E-22	ENSGALG00000029811	*BMPER*	234.06	2.26E-16	2.57E-15
ENSGALG00000042657	*Wnt3a*	22.57	5.00E-05	2.14E-04	ENSGALG00000028158	*FGF7*	224.87	5.40E-49	6.51E-47
ENSGALG00000002449	*DUOXA1L*	22.29	3.65E-16	4.02E-15	ENSGALG00000010331	*MME*	185.25	2.15E-33	9.53E-32
ENSGALG00000006322	*CLCA2*	21.99	4.01E-11	2.60E-10	ENSGALG00000026736	*OGN*	180.56	7.59E-64	2.43E-61
ENSGALG00000041822	*FGF22*	20.64	5.00E-05	2.14E-04	ENSGALG00000001071	*ELN*	178.38	1.08E-93	2.42E-90
ENSGALG00000009877	*KCNH1*	20.36	1.97E-23	4.09E-22	ENSGALG00000006877	*KLHL4*	175.81	4.72E-29	1.51E-27

### Validation of RNA-Seq data with qRT-PCR

**Figure 3:**
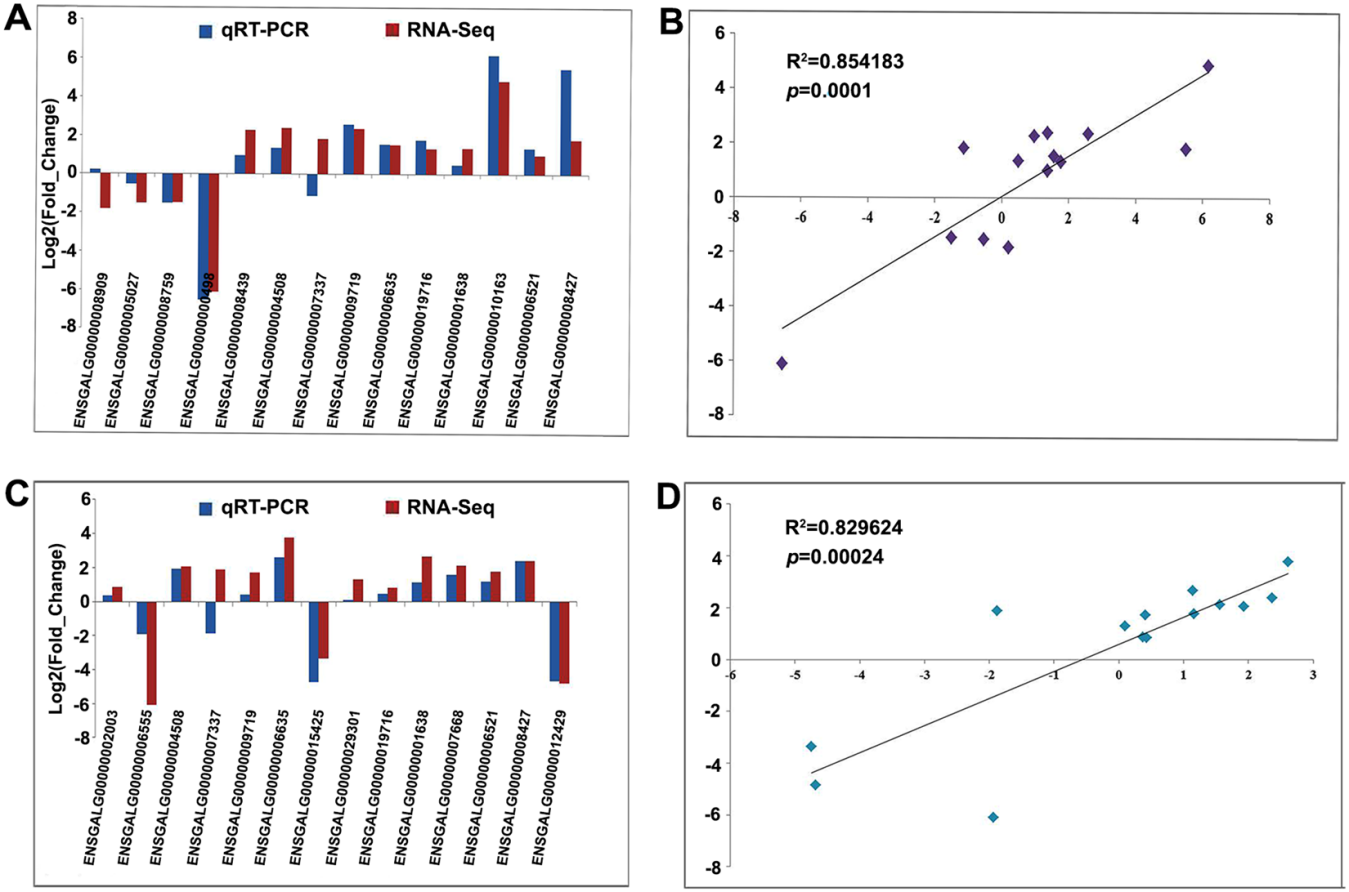
Correlation of expression levels of DEGs (20 in total, 8 in common in **A** and **C**) between GE vs NGE (**A,B**) or GE vs GM (**C**,**D**) detected by qRT-PCR and RNA-Seq. **A**, **C**: Histograms of expression levels of 14 selected DEGs between GE vs NGE (**A**) and GE vs GM (**C**). Y-axis represents the log2 (Fold_Change) derived from qRT-PCR and RNA-Seq. **B**, **D**: Regression analysis of the log2 (Fold_Change) values between RNA-Seq and qRT-PCR. The X- and Y-axis represents the log2 (Fold_Change) measured by qRT-PCR (normalized by GAPDH) and RNA-Seq, respectively.

To verify the accuracy of DEGs based on RNA-Seq and the aforementioned statistical criteria, qRT-PCR analysis was performed for 20 selected DEGs (|FC| > 1.5, *p* < 0.05 and FDR *q* < 0.05) (*DHX30*, *EYA2*, *LPP*, *PCDH10*, *RHCG*, *USP13*, *LAMP3*, *ACE*, *IGF2*, *LPL*, *CD36*, *KRT20*, *TAS1R3*, *LGR5*, *TRPM5*, *GNAT3*, *BMP2*, *BMP4*, *BMP7* and *Bmpr1a*) including highly and lowly expressed genes in the GE compared to NGE (6 DEGs) or GM (6 DEGs), and 8 DEGs (*KRT20*, *TAS1R3*, *TRPM5*, *GNAT3*, *RHCG*, *PCDH10*, *LPP*, *EYA2*) were shared DEGs by both GE vs NGE and GE vs GM comparisons. A comparison of expression of these 20 genes was made between the data from qRT-PCR normalized to *GAPDH* and those from RNA-Seq (Fig. 3). A high overlap is shown clearly by the concordant expression patterns between RNA-Seq and qRT-PCR and validates the adequacy of the statistical criteria used in these analyses. The computational and experimental fold changes in our study also showed a strong positive correlation (*p* < 0.001) with R^2^ = 0.854183 (*p* = 0.0001) in GE vs NGE (Fig. 3B) and R^2^ = 0.829624 (*p* = 0.00024) in GE vs GM (Fig. 3D) confirming the high reliability of RNA-Seq data in this study.

### Specific DEGs in the GE compared to surrounding tissue compartments (NGE and GM)

**Figure 4:**
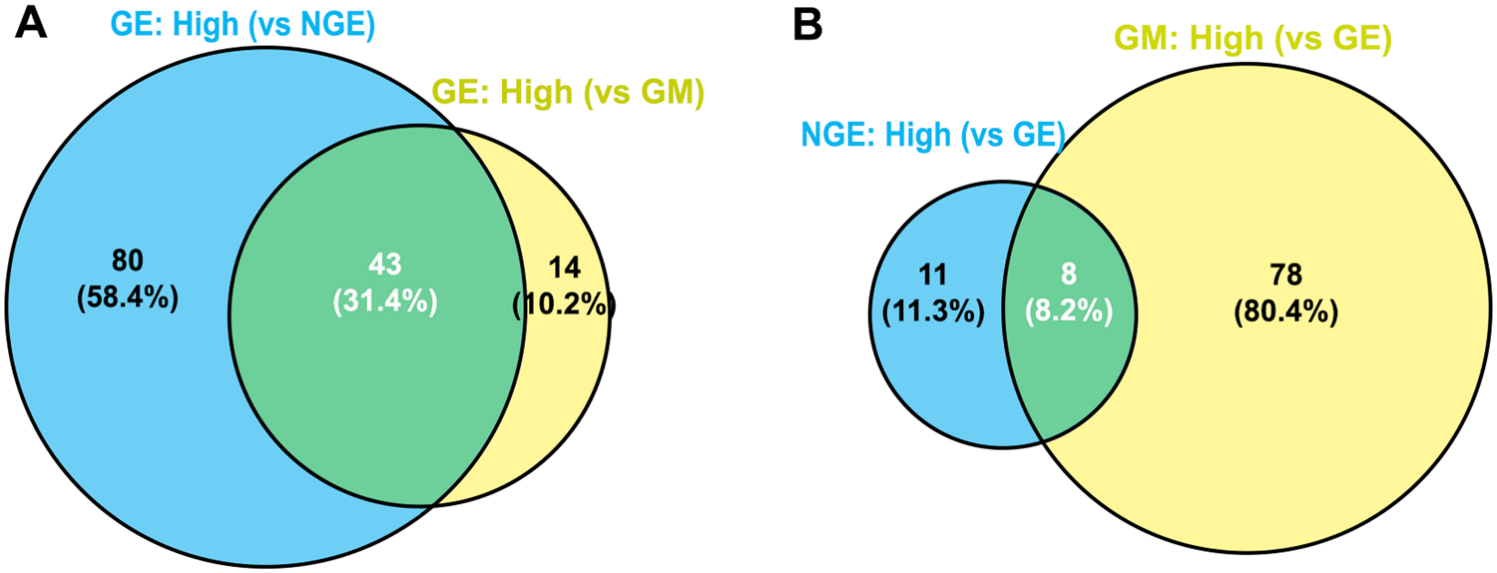
Venn diagram illustrating the number of distinct and overlapping DEGs in the GE compared to NGE and GM of the base of oral cavity. (**A**) Highly expressed DEGs in the GE compared with NGE (blue) and GM (yellow). (**B**) Highly expressed DEGs in the NGE and GM compared with GE. Statistically significant genes (*p* < 0.05) with a fold change** > **1.5 were included. GE: gustatory epithelium; NGE: non-gustatory epithelium; GM: gustatory mesenchyme.

To detect genes specific to the gustatory epithelium in which taste buds reside, we analyzed genes that are differentially expressed between GE and either of the two surrounding tissues (NGE and GM). Of the 143 DEGs identified, 51 genes were differentially expressed in the GE compared to both NGE and GM (43 at a higher and 8 at a lower level, |FC| > 1.5, *p* < 0.05 and FDR *q* < 0.05) (Fig. 4). This result suggests that these 51 genes (Table 3) are likely to be promising candidates that are particularly important for taste perception or regulation of taste organ development. Out of the 43 highly DEGs in the GE, 5 genes are already well-known to be important genes involved in the taste sensation and organogenesis (*GNAT3*, *TAS1R3*, *TRPM5*, *GNG13*, *SHH*).

**Table 3:** Fifty-one highly (43) or lowly (8) expressed transcripts in the GE compared to both NGE and GM.

Gene ID	Name	FC(NGE/GE)	FC(GM/GE)	*p*_value	*q_*value
ENSGALG00000016325	*GSTA3*	−19.46	−2.78**	6.03E-20	1.46E-16
ENSGALG00000035166	*LYPD2*	−9.02	−24.80**	5.00E-05	4.43E-03
ENSGALG00000133710	*SPINK5*	−5.68	−4.08**	5.00E-05	4.43E-03
ENSGALG00000004508	*EYA2*	−5.21	−3.87**	7.61E-13	4.92E-10
ENSGALG00000010901	*ABCB5*	−5.15	−2.54**	6.66E-05	6.73E-03
ENSGALG00000023303	*Six2*	−4.93	−3.64**	1.59E-07	3.96E-05
ENSGALG00000008185	*AOX1*	−4.30	−6.29**	5.00E-05	4.43E-03
ENSGALG00000009866	*CAMK1D*	−3.98	−3.31**	8.02E-06	1.17E-03
ENSGALG00000003553	*ABCA12*	−3.92	−1.94**	5.00E-05	4.43E-03
ENSGALG00000028598	*SERPINA12*	−3.75	−3.69**	1.43E-06	2.88E-04
ENSGALG00000014987	*OTOP1*	−3.65	−14.64**	7.33E-15	7.11E-12
ENSGALG00000007337	*LPP*	−3.55	−3.68**	5.00E-05	4.43E-03
ENSGALG00000008427	*GNAT3*	−3.48	−5.23*	4.78E-14	3.86E-11
ENSGALG00000020876	*AOX2*	−3.34	−7.63**	2.69E-17	4.34E-14
ENSGALG00000013033	*CMBL*	−3.19	−9.83**	1.43E-04	1.19E-02
ENSGALG00000006635	*RHCG*	−2.90	−12.68**	2.48E-09	1.15E-06
ENSGALG00000016522	*PPEF1*	−2.76	−16.23**	1.20E-07	3.07E-05
ENSGALG00000021232	*TGM6*	−2.73	−4.23**	6.34E-04	4.00E-02
ENSGALG00000003690	*KRT14*	−2.63	−11.68**	3.30E-09	1.39E-06
ENSGALG00000001638	*TAS1R3*	−2.58	−6.32**	1.65E-08	5.17E-06
ENSGALG00000007257	*TEKT5*	−2.56	−18.49**	3.44E-09	1.39E-06
ENSGALG00000017295	*PTHLH*	−2.53	−2.38**	7.31E-04	4.46E-02
ENSGALG00000019716	*KRT20*	−2.53	−1.78*	9.52E-09	3.37E-06
ENSGALG00000002024	*COMT*	−2.35	−1.95**	1.55E-06	3.07E-04
ENSGALG00000001617	*CAMKK1*	−2.13	−3.81**	2.82E-06	4.89E-04
ENSGALG00000014730	*ELOVL7*	−2.11	−2.42**	1.27E-04	1.08E-02
ENSGALG00000005293	*GNG13*	−2.03	−8.48*	1.31E-05	1.80E-03
ENSGALG00000023897	*RGS21*	−2.02	−11.64**	2.96E-06	5.03E-04
ENSGALG00000015593	*EPHA7*	−2.01	−3.35**	2.85E-05	3.41E-03
ENSGALG00000006521	*TRPM5*	−2.00	−3.37**	3.22E-05	3.67E-03
ENSGALG00000015764	*FABP5*	−1.98	−4.07**	6.00E-06	9.53E-04
ENSGALG00000016128	*B3GALT5*	−1.89	−4.78**	3.18E-05	3.67E-03
ENSGALG00000006379	*SHH*	−1.88	−3.82**	1.75E-04	1.41E-02
ENSGALG00000028946	*CSRP2*	−1.84	−3.37**	2.74E-06	4.89E-02
ENSGALG00000008105	*CYP2AB4*	−1.83	−6.08**	1.78E-04	1.43E-02
ENSGALG00000014372	*SLC34A2*	−1.83	−4.92**	3.07E-04	2.27E-02
ENSGALG00000040607	*MED23*	−1.79	−2.37**	2.50E-04	1.76E-02
ENSGALG00000003213	*LGALS2*	−1.74	−2.42**	5.99E-04	3.79E-02
ENSGALG00000011654	*ACPP*	−1.69	−7.12**	3.59E-04	2.58E-02
ENSGALG00000024272	*S100A9*	−1.68	−13.47**	6.95E-04	4.32E-02
ENSGALG00000016464	*VSNL1*	−1.64	−5.42**	1.41E-05	1.90E-03
ENSGALG00000006425	*SLC6A6*	−1.57	−2.06**	3.68E-05	4.00E-03
ENSGALG00000016324	*GSTA4*	−1.56	−4.01**	1.50E-04	1.24E-02
ENSGALG00000013145	*EPB41L4B*	1.75	1.59**	9.22E-05	8.61E-03
ENSGALG00000001527	*VWA1*	1.75	22.51**	4.83E-04	3.23E-02
ENSGALG00000005180	*COL11A1*	1.77	14.78**	1.05E-04	9.45E-03
ENSGALG00000026470	*BOD1L1*	1.81	2.17**	9.00E-04	4.90E-02
ENSGALG00000010763	*TRIP11*	1.94	2.08*	5.00E-05	4.43E-03
ENSGALG00000008399	*ANKRD12*	1.99	2.63**	6.00E-04	3.60E-02
ENSGALG00000009690	*CENPF*	2.15	2.29**	5.00E-05	4.43E-03
ENSGALG00000011687	*AHNAK2*	2.17	11.03**	3.00E-04	2.00E-02

*p < 0.05, **p < 0.01 for the expression of listed genes in the GE compared to both NGE and GM.

### Gene Ontology enrichment and pathway analysis

**Figure 5:**
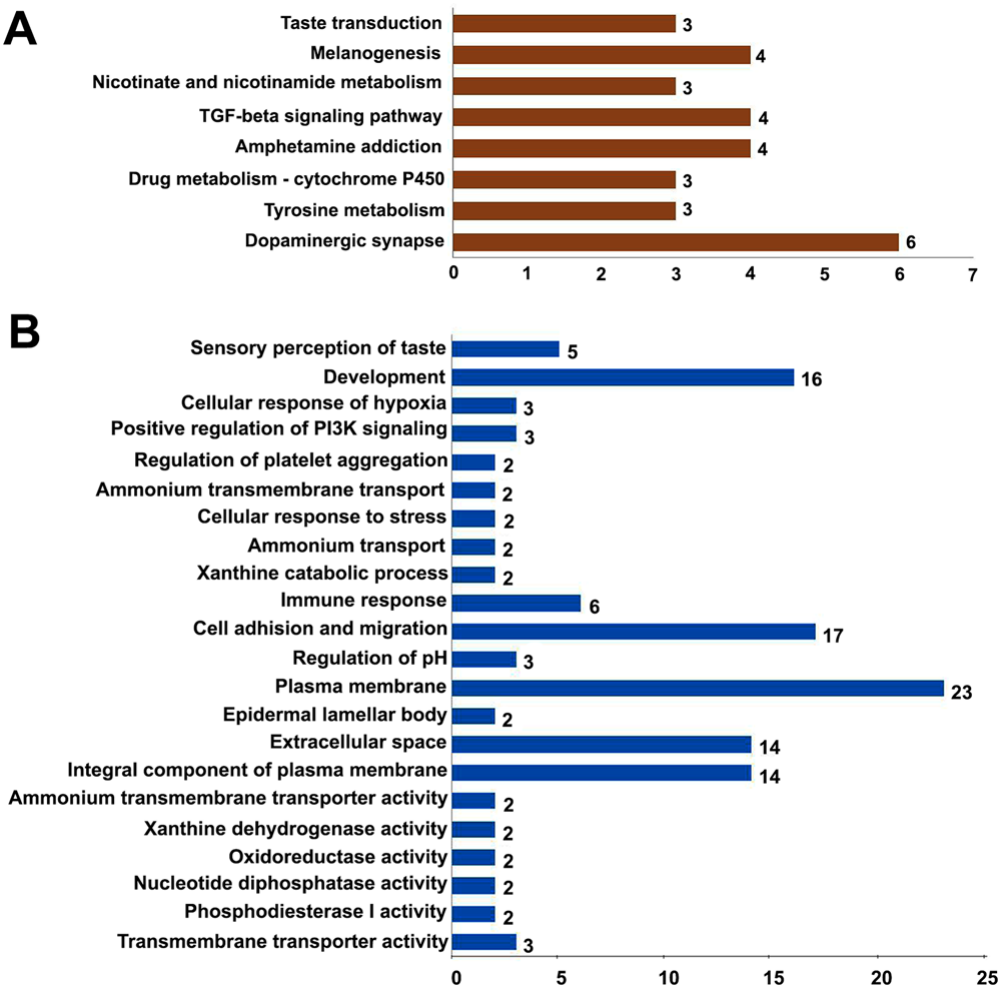
Histogram presentation of the number of DEGs between GE vs NGE of the base of oral cavity belonging to significant pathways (**A**) and selected gene ontology (GO) terms (**B**).

**Figure 6:**
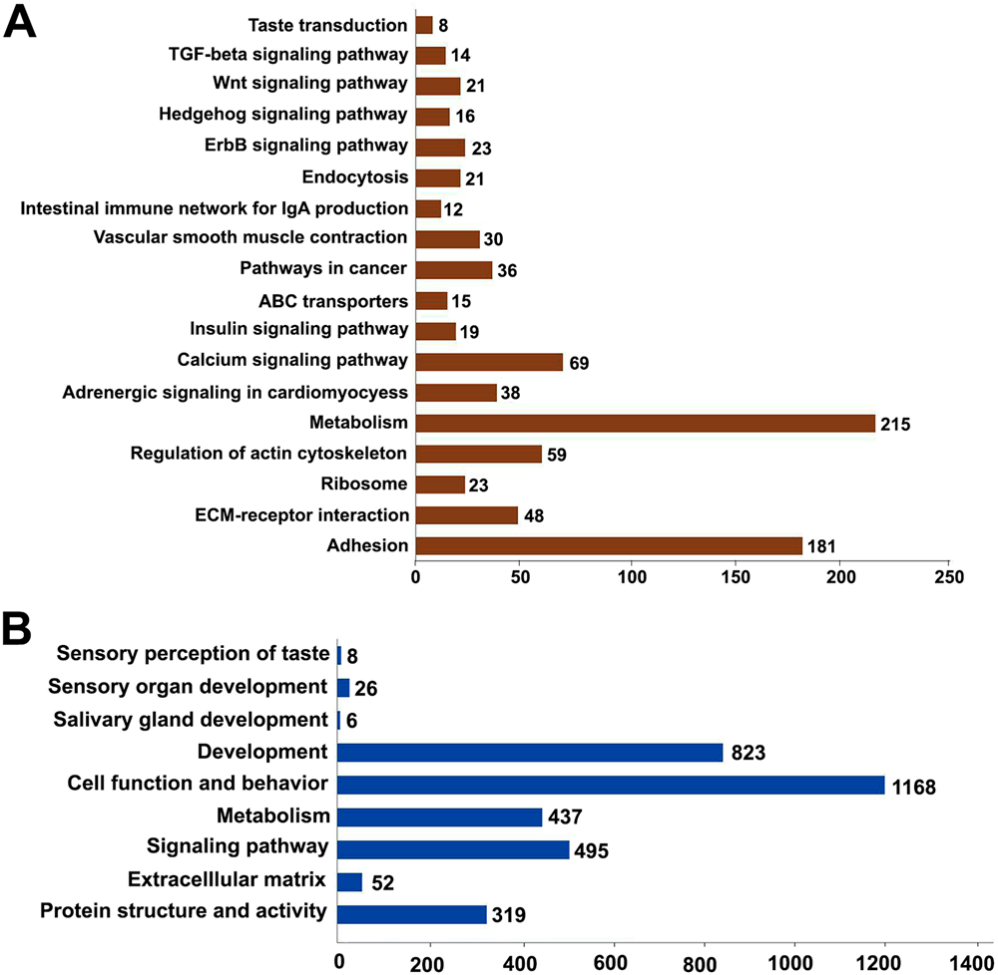
Histogram presentation of the number of DEGs between GE vs GM of the base of oral cavity belonging to significant pathways (**A**) and selected gene ontology (GO) terms (**B**).

To further investigate the functional associations of the DEGs, we performed gene ontology (GO) analysis at the GO database (http://www.geneontology.org/GO.database.shtml). The significance criteria was set at an unadjusted threshold at *p* < 0.05 and at least five DEGs in the background terms to assess potential functions. Multiple pathways and GO terms including biological processes, cellular components and molecular function were significantly enriched for these DEGs (Fig. 5 and 6). The details of the significant pathways in the two comparisons (GE vs NGE and GE vs GM) are shown in Supplemental Table S9, and the significant GO terms are shown in Supplemental Table S10.

From the comparison of GE vs NGE, 118 highly DEGs (|FC| > 2) were detected, among which 30 DEGs (Fig. 5A, Supplemental Table S9) were revealed to belong to significant pathways (8 in total) in the KEGG pathway analysis, including taste transduction, TGF-β signaling pathway, melanogenesis, dopaminergic synapse, drug metabolism-cytochrome P450, amphetamine addiction, nicotinate and nicotinamide metabolism, and tyrosine metabolism pathways (Fig. 5A, Supplemental Table S9). These 118 DEGs were classified into 32 significant GO categories (*p* < 0.05, Supplemental Table S10). Multiple GO terms including sensory perception of taste, development and other terms were significantly enriched for these DEGs as indicated in Fig. 5B. These DEGs include genes that are known to be involved in taste perception, e.g., *TAS1R3*, *GNG13*, *GNAT3*, and *CD36*, and genes that are important for the development of taste organs in rodents, such as *LGR5*, *TGFβ2*, *SHH*, and *LEF1* (Supplemental Table S10). Of note, some of the DEGs were shown to be involved in multiple functions (marked in red, Supplemental Table S10).

From the comparison of GE vs GM, 4313 highly DEGs (|FC| > 2) were detected and KEGG pathway analysis revealed 18 significant pathways (*p* < 0.05) with 848 gene profiles involved (Fig. 6A, Supplemental Table S9). The significant pathways include taste transduction, as well as TGF-β, hedgehog, and Wnt signaling pathways (Fig. 6A, Supplemental Table S9) that are well known and have been reported for their involvement in the development of tongue, taste papillae and taste buds^36–46^. These 4313 DEGs were classified into 378 significant GO categories (*p* < 0.05, Supplemental Table S10). The majority of these DEGs (3334) belong to 9 categories as shown in Fig. 6B. Similarly to the DEGs between GE vs NGE, many of the DEGs between GE vs GM were shown in multiple GO terms (Supplemental Table S10) indicating the involvement of these DEGs in multiple functions. Multiple GO terms including sensory perception of taste, sensory organ development, salivary gland development and development of other tissues were significantly enriched for these DEGs. Among the detected DEGs were *TRPM5*, *GNG13*, *TAS1R3*, *SCNN1G*, *SCNN1B*, *SCNN1A*, *PRKX* and *GNAT3*, which are known to be involved in the sensory perception of taste, and *LGR5*, *SHH*, *TGFβ2*, and *LEF1* (Supplemental Table S10), which are known to be involved in the development of taste organs.

**Figure 7:**
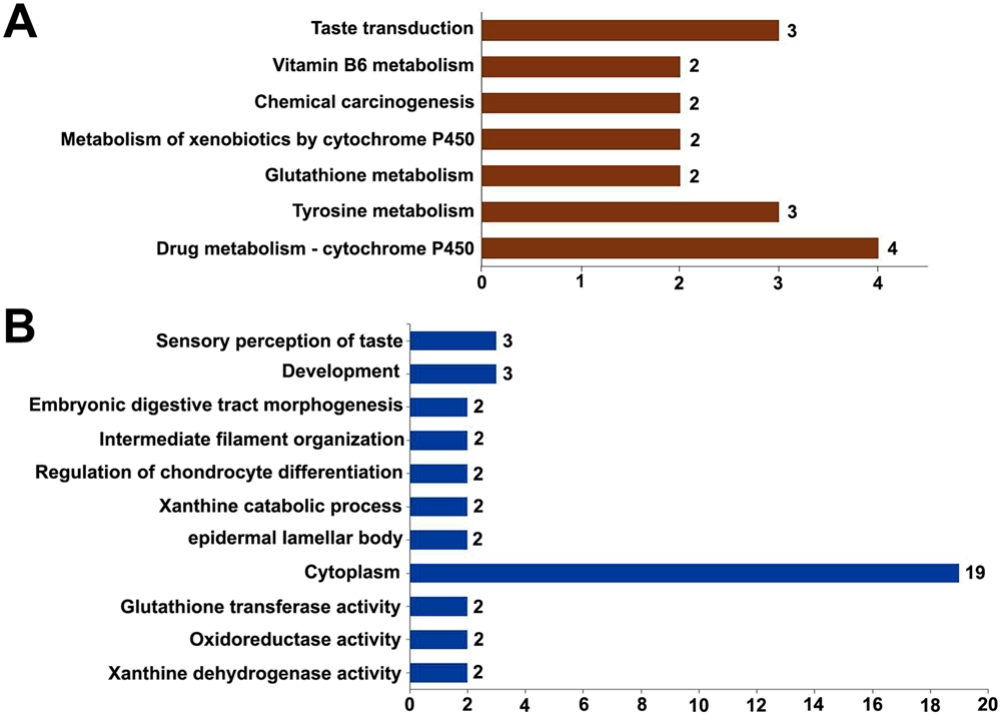
Histogram presentation of the number of shared DEGs belonging to significant pathways (**A**) and selected gene ontology (GO) terms (**B**). The shared DEGs were detected by comparisons of both GE vs NGE and GE vs GM of the base of oral cavity.

Among the 51 DEGs (Table 3), which were differentially expressed in the GE at a high (43) (Fig. 4A) or low (8) (Fig. 4B) level compared to both surrounding tissue compartments (NGE and GM), 18 gene profiles were found to fall into 7 significant KEGG pathways (Fig. 7A, Supplemental Table S9) and 46 gene profiles fall into 14 significant GO terms (Supplemental Table S10). Selected terms, as shown in Fig. 7B, include taste transduction pathway and sensory perception of taste.

### Mapping the expression of signaling components of molecular pathways in the gustatory epithelium and surrounding tissues

**Figure 8:**
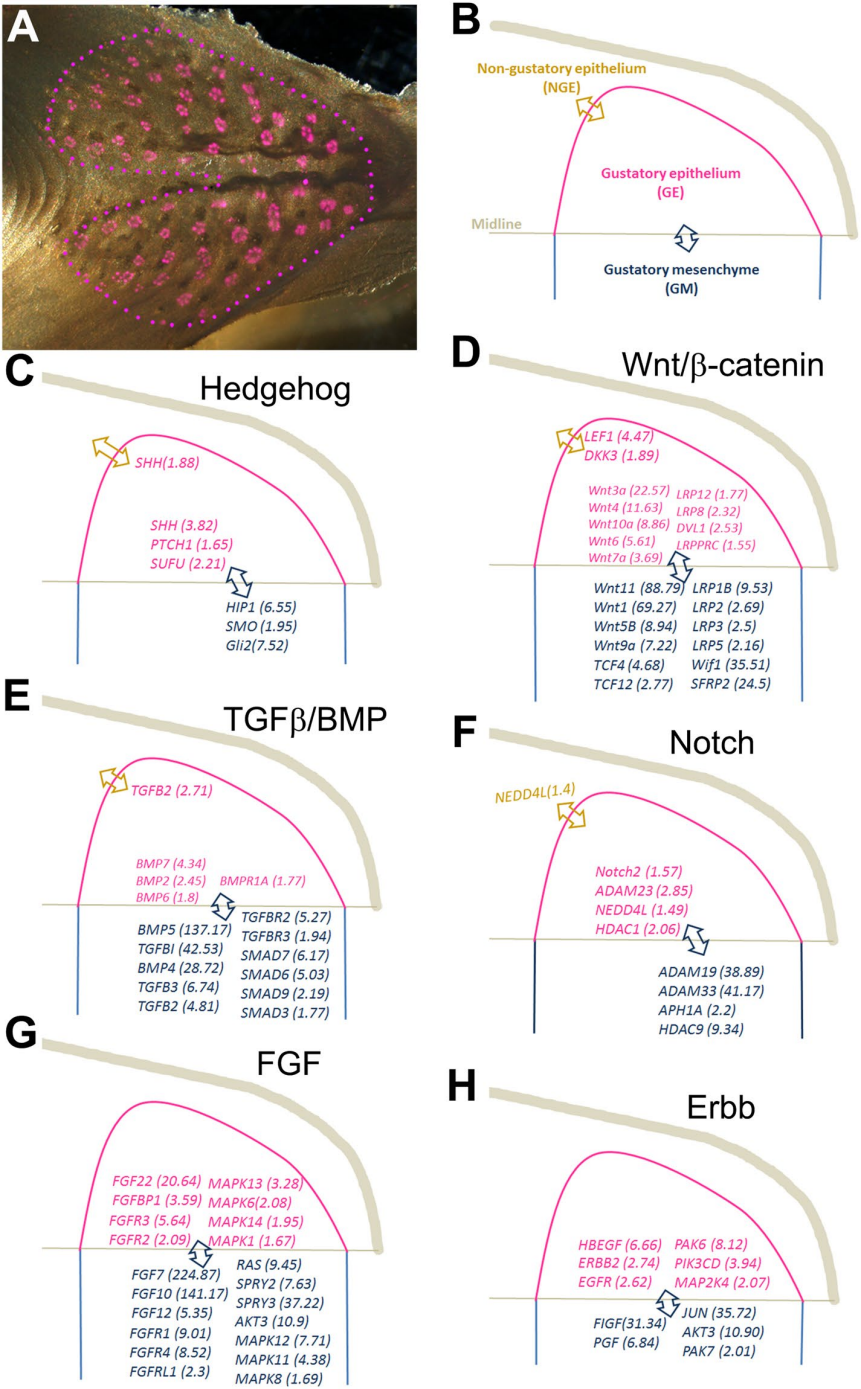
Distribution pattern of highly DEGs that are key components of well-known molecular signaling pathways in organogenesis (**C**–**H**). (**A**) An epithelial sheet of the base of the oral cavity of a P3 chicken that was immunoreacted with Vimentin for labeling taste buds (purple). Purple dots outline the gustatory epithelium that contains taste buds. (**B**) A diagram to illustrate the tissue compartments collected for RNA-Seq and DEG detection. (**B**–**H**) Double pointed arrows indicate the tissue compartments for comparisons of gene expressions. Values in parentheses in (**C**–**H**) represent the fold change of DEGs at a high expression level in the tissue compartment where the gene**’**s name is placed.

To better understand the potential function of molecular signaling pathways in regulating taste organ development in chickens, we selected several pathways that are known to be involved in taste organogenesis in rodents, e.g., Hedgehog^37, 41–43, 47–49^ (Fig. 8C), Wnt/β-catenin^38, 40, 50–52^ (Fig. 8D), TGF-β/BMP^36, 46, 53, 54^ (Fig. 8E), Notch^55–57^ (Fig. 8F), fibroblast growth factor (FGF) ^53, 58, 59^ (Fig. 8G), and Erbb^60–62^ (Fig. 8H), and schematically illustrated the spatial distribution of highly expressed key components in gustatory epithelium (GE), non-gustatory epithelium (NGE), and gustatory mesenchyme (GM).

Some key components were found to be highly expressed in the GE compared to the NGE, e.g., *SHH* of the hedgehog signaling pathway (Fig. 8C), *LEF1* and *DKK3* of the Wnt/β-catenin signaling pathway (Fig. 8D), *TGFβ2* of the TGFβ/BMP signaling pathway (Fig. 8E). An ubiquitin ligase neural precursor cell expressed developmentally downregulated gene 4-like (*NEDD4L*) was found to be expressed in the gradient of NGE > GE > GM (Fig. 8F).

From the comparison between GE and GM, a large number of signaling components in each of the aforementioned signaling pathways were identified as DEGs (Supplemental Table S8), including ligands, receptors, intracellular signaling components, and transcription factors. Some key components were selected and presented in Fig. 8C-H. The gene expression patterns suggest their potentially active involvement in the epithelial-mesenchymal interactions in the gustatory tissue.

## Discussion

RNA sequencing (RNA-Seq) is currently the most powerful tool available to study the complexity of the transcriptome. It has many advantages over traditional cDNA microarray technologies, including issues related to probe design hybridization bias^63^ and sensitivity to detect low-abundance transcripts^64^. To identify candidate genes involved in the development of taste organs, we used RNA-Seq and investigated the transcriptomic profile of gustatory tissue and its surrounding compartments found in the chicken oral cavity.

In our analysis, we minimized false-positives and ensured substantial detection power and accuracy using several strategies to detect DEGs between samples by controlling critical influencing factors. Analyses were performed on deep-sequenced transcripts using two commonly used packages: DESeq2 and Cuffdiff, to ensure a higher sensitivity of algorithms that control FDR^65^ and provide greater inferential power in a typical RNA-Seq experiment with small replicate numbers^4, 66, 67^, and a rigorous statistical analysis^32^.

Furthermore, we confirmed the RNA-Seq results using qRT-PCR assay for a selected subset of DEGs. Overall, there was a high concordance and strong positive correlation between statistical and experimental results, revealing a high detection sensitivity and accuracy, which is similar to previous reports in animals^9, 14, 16, 68, 69^.

The mammalian taste organs including the tongue, taste papillae, and taste buds have been an ideal system for organogenesis studies and have attracted numerous researchers seeking to advance the field of developmental biology^70–72^. The topographic distribution of taste papillae and taste buds asserts taste organs as an ideal system for studying pattern formation^73^. Chicken taste buds, like those of mammals, are distributed in a patterned array^28^ and the many large clusters enables us to collect enough tissue for different analyses conveniently and efficiently. In the present study, our findings using RNA-Seq analysis provide useful information for further functional studies in organogenesis and regenerative medicine.

The functional enrichment analyses showed that multiple KEGG pathways and most GO terms are rigorously involved in sensory perception of taste, as well as the development of sensory organs and salivary glands. These results indicated that the identified DEGs may play important roles in taste function and taste organ development. Combining the significant number and expression levels of DEGs, GO and pathway results, and gene function, allows us to suggest that *GNG13*, *SCNN1A*, *SCNN1B*, *SCNN1G* and *PRKX* as 5 promising candidate genes for sensory perception of taste in chickens. In addition, 3 known genes (*GNAT3*, *TRPM5* and *TAS1R3*) were revealed to be involved in taste perception and 4 analogous genes (*SHH*, *TGFβ2*, *DKK3* and *LEF1*) are known to be involved in the development of taste organs in mouse. A large subset of key components of well-known molecular signaling pathways (Hedgehog, Wnt/β-catenin, TGFβ/BMP, Notch, FGF, Erbb) that are important for organogenesis in rodents were found to be highly DEGs between GE and GM. This suggests a potentially active involvement of these signaling pathways in the epithelial-mesenchymal interactions that are required for organogenesis^74^.

Of note, DEGs between GE and GM were also found to be enriched in salivary gland development. Chicken taste buds are often clustered in areas surrounding salivary gland openings^24, 28^. It is reasonable to speculate that the development of taste buds and salivary glands in chickens are likely to be associated or linked. Further studies on whether, what and how signaling molecules coordinate the organogenesis of taste buds and salivary glands openings will be beneficial for better understanding the formation of both organs.

In summary, chicken taste buds are distributed in a unique pattern in the gustatory tissue of the oral cavity. They have a much shorter lifespan (~4 days)^75, 76^ compared to mammals (~10–12 days in rodents)^77, 78^ indicating a more active progenitor cell niche and allowing for a more efficient way to study taste bud cell renewal compared to the rodent model. Moreover, the connective tissue cell marker Vimentin is expressed in a large population of taste bud cells in chickens, similar to humans^79, 80^, suggesting a comparable mechanism underlying the contribution of connective tissue to taste buds in both organisms. Furthermore, new taste buds continue to develop after hatching in female-line male chickens^28^, which provides a time window to study the regulation of taste bud development. In combination with other beneficial aspects of using chickens as a research model, e.g., convenience of *in ovo* embryo manipulation, high availability and rapid development^81, 82^, we propose that the chicken taste organ can serve as an ideal model for studies in organogenesis and regenerative medicine.

## Electronic supplementary material

Supplemental Table S1

Supplemental Table S2

Supplemental Table S3

Supplemental Dataset 1

Supplemental Dataset 2

Supplemental Dataset 3

Supplemental Dataset 4

Supplemental Dataset 5

Supplemental Dataset 6

Supplemental Dataset 7
