# Supplementary material for: RNA-Seq analysis on chicken taste sensory organs: An ideal system to study organogenesis
Source: Sci Rep. 2017 Aug 22;7:9131. doi: 10.1038/s41598-017-09299-7 (PMC5567234; doi:10.1038/s41598-017-09299-7)
Supplement: Supplementary file 1 — Supplemental Table S1 [file 41598_2017_9299_MOESM1_ESM.pdf]

RNA-Seq analysis on chicken taste sensory organs:

An ideal system to study organogenesis

Xiaogang Cui[1, 2], Brett Marshall[1, 2], Ning Shi[3], Shi-You Chen[3], Romdhane Rekaya[2, 4], Hong-Xiang Liu[1, 2] *

[1] Regenerative Bioscience Center, University of Georgia, Athens, GA, USA

[2] Department of Animal and Dairy Science, College of Agricultural and Environmental Sciences, University of Georgia, Athens, GA, USA

[3] Department of Physiology and Pharmacology, College of Veterinary Medicine, University of Georgia, Athens, GA, USA

[4] Institute of Bioinformatics, University of Georgia, Athens, GA, USA

Running title: RNA-Seq analysis in chicken taste organs

*Correspondence should be sent to: Hong-Xiang Liu, Regenerative Bioscience Center, Department of Animal and Dairy Science, College of Agricultural and Environmental Sciences, University of Georgia, 425 River Road, Athens, GA 30602, USA. Email: lhx@uga.edu.

Supplemental Table S1. Summary statistics for sequence quality and alignment information of nine samples.

Statistic	GE1	GE2	GE3	GM1	GM2	GM3	NGE1	NGE2	NGE3
Raw Reads	59,946,034	42,835,845	36,119,850	30,189,852	35,937,806	21,678,518	33,873,435	40,615,590	34,460,014
Clean Reads	58,147,653	41,550,770	35,036,254	29,284,156	34,859,672	21,028,162	32,857,232	39,397,122	33,426,214
Q20 (%)	94.75	96.59	95.48	96.71	94.99	96.41	95.23	96.57	94.57
Q30 (%)	90.08	90.06	90.12	90.3	90.21	89.94	90.25	90.04	89.84
GC content (%)	46.31	47.7	48.6	46.98	47.78	48.95	46.35	47.45	48.38
Total mapped reads	33,193,001	34,626,935	28,133,701	25,009,799	28,798,643	17,431,655	27,508,537	32,613,280	26,821,995
Unique mapped reads	32,761,270	34,038,694	27,491,354	24,590,659	27,858,349	16,831,061	27,097,869	32,000,075	26,188,443
Multiple mapped reads	431,509	588,658	647,075	425,167	921,556	592,676	412,628	587,039	616,906
Spliced mapped reads	8,449,099	8,941,284	6,932,976	6,946,082	7,308,376	4,756,427	6,731,585	8,166,181	6,801,035
Mapping Rate (%)	85.65	83.34	80.36	85.48	82.62	82.93	83.74	82.86	80.26
