# Supplementary material for: RNA-Seq analysis on chicken taste sensory organs: An ideal system to study organogenesis
Source: Sci Rep. 2017 Aug 22;7:9131. doi: 10.1038/s41598-017-09299-7 (PMC5567234; doi:10.1038/s41598-017-09299-7)
Supplement: Supplementary file 2 — Supplemental Table S2 [file 41598_2017_9299_MOESM2_ESM.pdf]

RNA-Seq analysis on chicken taste sensory organs:

An ideal system to study organogenesis

Xiaogang Cui[1, 2], Brett Marshall[1, 2], Ning Shi[3], Shi-You Chen[3], Romdhane Rekaya[2, 4], Hong-Xiang Liu[1, 2]*

[1] Regenerative Bioscience Center, University of Georgia, Athens, GA, USA

[2] Department of Animal and Dairy Science, College of Agricultural and Environmental Sciences, University of Georgia, Athens, GA, USA

[3] Department of Physiology and Pharmacology, College of Veterinary Medicine, University of Georgia, Athens, GA, USA

[4] Institute of Bioinformatics, University of Georgia, Athens, GA, USA

Running title: RNA-Seq analysis in chicken taste organs

*Correspondence should be sent to: Hong-Xiang Liu, Regenerative Bioscience Center, Department of Animal and Dairy Science, College of Agricultural and Environmental Sciences, University of Georgia, 425 River Road, Athens, GA 30602, USA. Email: lhx@uga.edu.

Supplemental Table S2, Primers information and validation results of the 14 chosen differentially expressed genes (GE vs NGE) by qRT-PCR analysis.

Gene Name	Log2 FC by RNA-Seq	Log2 FC by qRT-PCR	Forward primer sequence	Reverse primer Sequence	Amplicon (bp)
USP13	-1.81	0.22	AGAAGGTACCAGGGGCATCT	TCACCAGTGCTGGTAACTCTTC	189
DHX30	-1.52	-0.53	AGAACCAGGTGGGATCCTCT	CGCTGGAAGATGTTCTGTTG	159
LAMP3	-1.47	-1.5	ATTCGCCAGCAGCATTTTAC	CGGGAACTGCCTTTGTACTT	190
ACE	-6.11	-6.54	CGGATCAAGGAGGACGAATA	CTGGATCACGAAGCTGACAA	168
CD36	2.27	0.98	TGCAATTTGCCAAAAGACTG	CCGACCGGTGACTTTATTTC	195
EYA2	2.38	1.37	ACGGCATACCTTCCTACAGC	ATTGGCTCCCTGGTAAATCC	164
LPP	1.83	-1.12	TTGAAAATCGGTGGGAACAT	CACTGGACATGGAAGTCACG	152
PCDH10	2.36	2.59	AAAATTCCAACGGCAGCAT	GCTCACTGTCTCCATGACCA	153
RHCG	1.53	1.56	ATCCTCCTCAACCTGCTTCA	GGGCCAGTACATCCACAGAT	198
GNAT3	5.5	1.8	GGTGCTGGAGAGTCAGGAAA	CAATTCTAGCGGGGTTTTCA	187
TRPM5	1.37	1	GACAATGGGGCCAACATAAC	TCTCCTGCTGTTGACCAGTG	157
TAS1R3	1.37	0.51	TGGGTGCTGCTGTGTATGAG	TACTGTCAGCTGTGCCCAAG	237
LGR5	4.84	6.18	CAAAGCAGACACATTCCGGC	GTAGCCCCGTCACAGGAAAA	161
KRT20	1.34	1.78	AGCTTGTCCTGCAGATCGAC	GCAGTCTGTCAACCTCCTCC	228
GAPDH as control			CGTCCTCTCTGGCAAAGTCC	TTCCCGTTCTCAGCCTTGAC	

Abbreviations: FC=fold change
