# Supplementary material for: RNA-Seq analysis on chicken taste sensory organs: An ideal system to study organogenesis
Source: Sci Rep. 2017 Aug 22;7:9131. doi: 10.1038/s41598-017-09299-7 (PMC5567234; doi:10.1038/s41598-017-09299-7)
Supplement: Supplementary file 3 — Supplemental Table S3 [file 41598_2017_9299_MOESM3_ESM.pdf]

RNA-Seq analysis on chicken taste sensory organs:

An ideal system to study organogenesis

Xiaogang Cui[1, 2], Brett Marshall[1, 2], Ning Shi[3], Shi-You Chen[3], Romdhane Rekaya[2, 4], Hong-Xiang Liu[1, 2] *

[1] Regenerative Bioscience Center, University of Georgia, Athens, GA, USA

[2] Department of Animal and Dairy Science, College of Agricultural and Environmental Sciences, University of Georgia, Athens, GA, USA

[3] Department of Physiology and Pharmacology, College of Veterinary Medicine, University of Georgia, Athens, GA, USA

[4] Institute of Bioinformatics, University of Georgia, Athens, GA, USA

Running title: RNA-Seq analysis in chicken taste organs

*Correspondence should be sent to: Hong-Xiang Liu, Regenerative Bioscience Center, Department of Animal and Dairy Science, College of Agricultural and Environmental Sciences, University of Georgia, 425 River Road, Athens, GA 30602, USA. Email: lhx@uga.edu.

Supplemental Table S3, Primers information and validation results of the 14 chosen differentially expressed genes (GE vs GM) by qRT-PCR analysis.

Gene Name	Log2 FC by RNA-Seq	Log2 FC by qRT-PCR	Forward primer sequence	Reverse primer Sequence	Amplicon (bp)
IGF2	-6.09	-1.94	TGTGGAGACAGAGGCTTCTACTT	CACGTACAGAGCGTGCAGAT	172
EYA2	2.04	1.93	ACGGCATACCTTCCTACAGC	ATTGGCTCCCTGGTAAATCC	164
LPP	1.88	-1.88	TTGAAAATCGGTGGGAACAT	CACTGGACATGGAAGTCACG	152
PCDH10	1.71	0.41	AAAATTCCAACGGCAGCAT	GCTCACTGTCTCCATGACCA	153
RHCG	3.76	2.61	ATCCTCCTCAACCTGCTTCA	GGGCCAGTACATCCACAGAT	198
LPL	-3.35	-4.75	GCTGGTCCCACCTTTGAGTA	GCTGGAAACCTCCACCATTA	169
GNAT3	2.39	2.37	GGTGCTGGAGAGTCAGGAAA	CAATTCTAGCGGGGTTTTCA	187
TRPM5	1.75	1.16	GACAATGGGGCCAACATAAC	TCTCCTGCTGTTGACCAGTG	157
TAS1R3	2.66	1.14	TGGGTGCTGCTGTGTATGAG	TACTGTCAGCTGTGCCCAAG	234
KRT20	0.83	0.43	AGCTTGTCCTGCAGATCGAC	GCAGTCTGTCAACCTCCTCC	228
BMP2	1.29	0.11	AACAGCAGCTACCATCACCG	GTGAACCACCTCCACCACAA	207
BMP4	-4.84	-4.69	TCCGCTTCGTCTTCAACCTC	AGCGGCTTCATCACTTCGTA	151
BMP7	2.12	1.56	TCACGGTCGCCTTCTTCAAA	TAGCCCTCTGGAGCGATGAT	235
Bmpr1a	0.87	0.37	GGACTTTTTGATGGCAGCAT	TCATCTTGTTCCAGGTCACG	158
GAPDH as control			CGTCCTCTCTGGCAAAGTCC	TTCCCGTTCTCAGCCTTGAC	

Abbreviations: FC=fold change
